# Spatial transcriptomics identifies candidate stromal drivers of benign prostatic hyperplasia

**DOI:** 10.1172/jci.insight.176479

**Published:** 2024-01-23

**Authors:** Anna S. Pollack, Christian A. Kunder, Noah Brazer, Zhewei Shen, Sushama Varma, Robert B. West, Gerald R. Cunha, Laurence S. Baskin, James D. Brooks, Jonathan R. Pollack

**Affiliations:** 1 Department of Pathology, Stanford University School of Medicine, Stanford, California, USA.; 2 Department of Urology, University of California, San Francisco (UCSF), San Francisco, California, USA.; 3 Department of Urology, Stanford University School of Medicine, Stanford, California, USA.

**Keywords:** Cell Biology, Reproductive Biology, Expression profiling, Molecular pathology, Urology

## Abstract

Benign prostatic hyperplasia (BPH) is the nodular proliferation of the prostate transition zone in older men, leading to urinary storage and voiding problems that can be recalcitrant to therapy. Decades ago, John McNeal proposed that BPH originates with the “reawakening” of embryonic inductive activity by adult prostate stroma, which spurs new ductal proliferation and branching morphogenesis. Here, by laser microdissection and transcriptional profiling of the BPH stroma adjacent to hyperplastic branching ducts, we identified secreted factors likely mediating stromal induction of prostate glandular epithelium and coinciding processes. The top stromal factors were insulin-like growth factor 1 (*IGF1*) and CXC chemokine ligand 13 (*CXCL13*), which we verified by RNA in situ hybridization to be coexpressed in BPH fibroblasts, along with their cognate receptors (*IGF1R* and *CXCR5*) on adjacent epithelium. In contrast, *IGF1* but not *CXCL13* was expressed in human embryonic prostate stroma. Finally, we demonstrated that IGF1 is necessary for the generation of BPH-1 cell spheroids and patient-derived BPH cell organoids in 3D culture. Our findings partially support historic speculations on the etiology of BPH and provide what we believe to be new molecular targets for rational therapies directed against the underlying processes driving BPH.

## Abstract 2

<p>Spatial transcriptomic analysis of benign prostatic hyperplasia (BPH) nodules uncovers stromal drivers of BPH pathogenesis that inform disease etiology and suggest potential avenues for disease-targeting therapy.</p>

## Graphical abstract

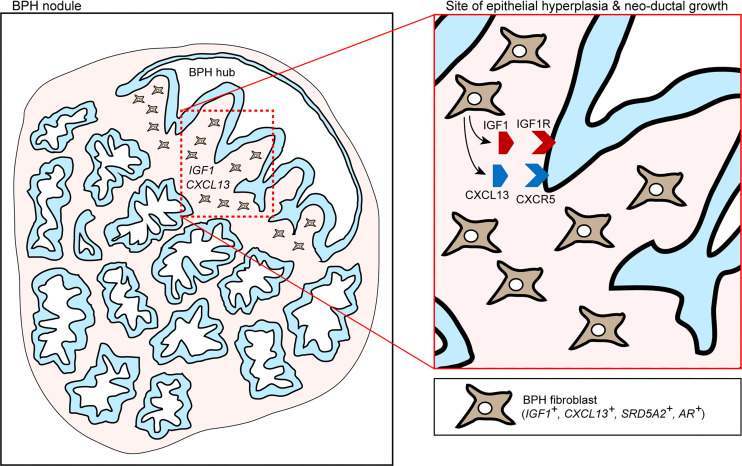

## Introduction

Benign prostatic hyperplasia (BPH) is the nodular proliferation of epithelial and stromal elements within the transition zone of the prostate (1). BPH leads to prostate enlargement and lower urinary tract symptoms — including urinary storage and voiding problems — that cause substantial morbidity in men over 50 years old (1). Current BPH treatments — including α-adrenoceptor antagonists and 5α-reductase inhibitors — are only partially effective and may not target the underlying pathologic processes, which are still incompletely known.

**Figure 1:**
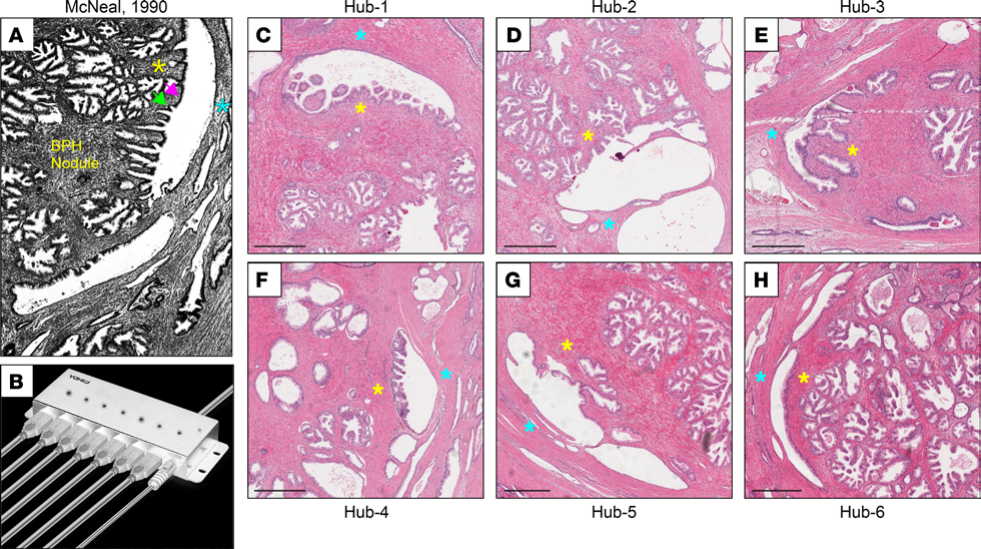
“BPH hubs” suggest an inductive stroma. (**A**) Tangent ducts at the periphery of BPH nodules display epithelial hyperplasia (with taller columnar epithelial cells) (pink arrow) and neoductal growth (green arrow) exclusively on the side facing into the nodule, implying an inductive inner stroma (yellow star) and noninductive outer stroma (blue star). Histologic image from J McNeal in *The Urologic Clinics of North America* (3) with with permission of the publisher. (**B**) For shorthand, we term these ductal structures “BPH hubs” for their similarity to a wagon wheel or USB hub (shown). (**C**–**H**) Six hubs (Hubs 1–6) that display the classic features noted by McNeal were selected for laser capture microdissection. Inner stroma (yellow star) and outer stroma (blue star) are indicated. Scale bar is 500 μm.

In the 1970s, Stanford pathologist John McNeal recorded detailed observations of BPH histology that informed a new hypothesis of BPH etiology (2, 3). He noted that the tangent ducts bordering BPH nodules exhibited epithelial hyperplasia and neoductal growth exclusively on the side facing *into* the nodule (Figure 1A). This eccentric effect suggested exposure to stromal inductive processes known to occur in prostate embryogenesis and later shown to be critical to epithelial and duct growth by tissue recombination studies (4). Thus, McNeal proposed that BPH nodules arise by the “reawakening” of embryonic inductive activity by adult prostate stroma. Whether the diffusible factors of BPH stroma mirror those of prostate embryogenesis has remained largely unknown.

Studies of gene-knockout mice and of rat prostatic rudiments in culture have identified secreted factors that modulate prostate development and ductal branching morphogenesis (5, 6), including fibroblast growth factors (FGFs), insulin-like growth factors (IGFs), transforming growth factors (TGFs), bone morphogenic proteins (BMPs), WNT proteins (WNTs), and Sonic Hedgehog (SHH). By extension, many of those same factors have been implicated in BPH (7). More recently, bulk tissue and single-cell transcriptome studies have enumerated the genes expressed in human BPH tissues and cells (8, 9) but have lacked histologic spatial resolution.

Here, by spatial transcriptomics of human BPH nodules, we identify secreted factors expressed in the presumptive inductive stroma adjacent to hyperplastic branching ducts. Our findings provide insight into McNeal’s hypothesis of embryonic reawakening and suggest what we believe to be new avenues for disease-targeted therapy.

## Results

### Spatial transcriptomics identifies factors secreted by presumptive inductive BPH stroma.

McNeal observed that tangent ducts bordering BPH nodules exhibit epithelial hyperplasia (with taller columnar epithelial cells) and neoductal growth exclusively on the side facing into the nodule (Figure 1A) (3), suggestive of inductive signals originating in the adjacent stroma. For shorthand, we have dubbed these branching ductal structures “BPH hubs” because of their superficial resemblance to a wagon wheel or USB hub (Figure 1B).

**Figure 2:**
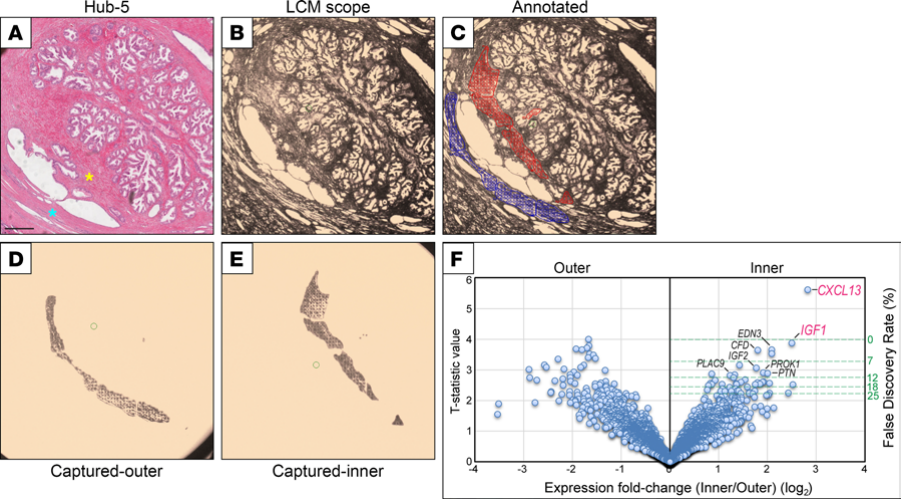
Spatial transcriptomics of BPH hubs nominates stromal inductive factors. (**A**–**E**) Laser capture microdissection (LCM) exemplified for Hub-5. (**A**) H&E stain. Inner stroma (yellow star) and outer stroma (blue star) are indicated; scale bar is 500 μm. Note, the H&E section shown in **A** is the same as that presented in Figure 1G. (**B**) Corresponding LCM scope image. (**C**) Annotated LCM scope image. The red (inner stroma) and blue (outer stroma) markings register spots for infrared welding laser and boundaries for ultraviolet cutting laser. (**D**) Captured outer stroma. (**E**) Captured inner stroma. (**F**) Volcano plot of RNA-Seq–identified gene transcripts significantly overexpressed in the inner versus outer stroma of the 6 microdissected hubs. FDR is plotted against log_2_ expression fold-change. Select genes significantly overexpressed in the inner (inductive) stroma are indicated.

To investigate McNeal’s hypothesis, we first sought to profile and compare gene expression in the “inner” (inside the nodule) versus “outer” adjacent stroma of BPH hubs. By examining H&E-stained sections of whole-mount human prostates (obtained from patients with prostate cancer and mild to severe BPH symptoms), we identified 6 BPH hubs with the classic features described by McNeal (Hubs 1–6 in Figure 1, C–H, and Supplemental Table 1; supplemental material available online with this article; https://doi.org/10.1172/jci.insight.176479DS1). Combining laser capture microdissection (LCM) (Figure 2, A–E) and RNASeq, we generated paired spatial transcriptomes of the 6 BPH hubs. By supervised analysis of the gene expression data, we then identified genes whose expression was significantly enriched in the inner or outer stroma of BPH hubs (Figure 2F and Supplemental Table 2). Here, we focus on transcripts enriched in the inner stroma, which would include candidate inductive factors.

The topmost gene transcripts enriched in the inner stroma were insulin-like growth factor 1 (*IGF1*) and CXC chemokine ligand 13 (*CXCL13*). IGF1 has previously been linked to prostate development (10) and has been implicated, among many other growth factors, in BPH pathogenesis (7). CXCL13 is a lymphocyte chemoattractant (11) that we recently found overexpressed in bulk BPH tissue (8). Other top transcripts enriched in the inner stroma include endothelin 3 (*EDN3*), complement factor D (*CFD*), prostaglandin D_2_ synthase (*PTGDS*), insulin-like growth factor 2 (*IGF2*), prokineticin 1 (*PROK1*), pleiotrophin (*PTN*), and placenta associated 9 (*PLAC9*). Notably, 8 of the top 10 genes (80%), and 11 of the top 20 (55%) genes, are known or predicted secreted proteins, representing a significant enrichment over the known human proteome (9.4%) (*P* ≤ 3.7 × 10^–7^; Hypergeometric test) (12). By Ingenuity Pathway Analysis (IPA; QIAGEN), the top inner stromal genes were enriched for physiologic processes that included tissue development, tissue morphology, and organ morphology (Supplemental Table 3). Our subsequent studies focused on the 2 top genes, *IGF1* and *CXCL13*.

**Figure 3:**
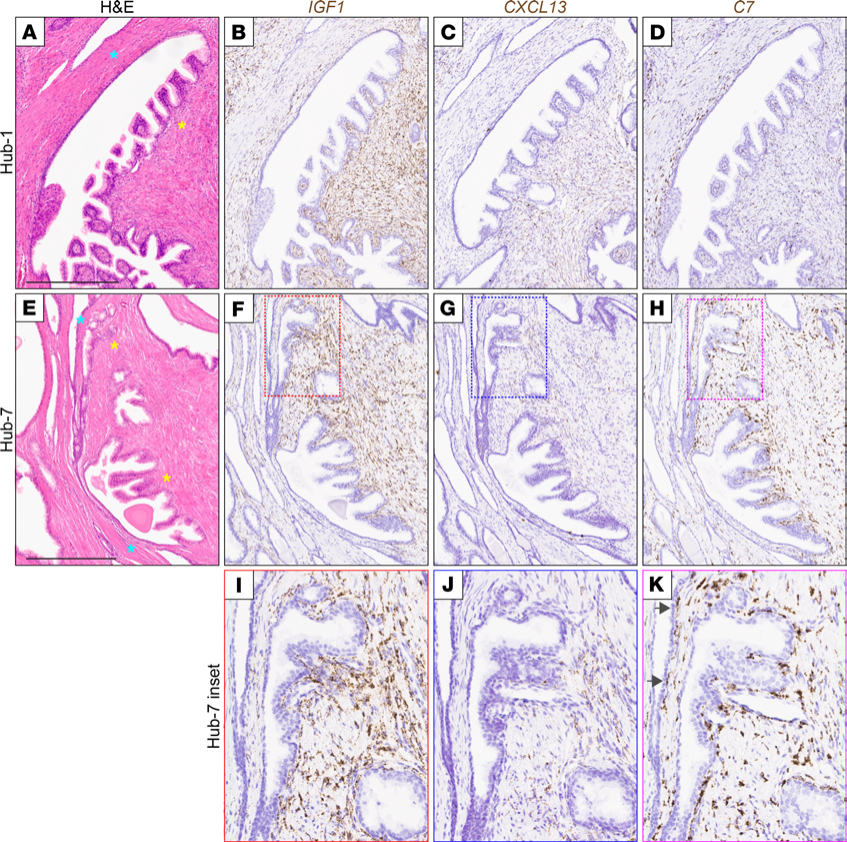
RISH verifies *IGF1* and *CXCL13* expression in inner (inductive) stroma. (**A**–**D**) RISH exemplified for a laser-microdissected hub, Hub-1. (**E**–**K**) RISH exemplified for an independent hub, Hub-7. (**A** and **E**) H&E stain. Inner stroma (yellow star) and outer stroma (blue star) are indicated; scale bar is 500 μm, and all figure insets are an additional 2.7× magnification. (**B**, **F**, and **I**) *IGF1* expression by RISH (brown staining). (**C**, **G**, and **J**) *CXCL13* expression by RISH (brown staining). (**D**, **H**, and **K**) *C7* expression (marks interstitial prostate fibroblasts) by RISH (brown staining); arrows indicate fibroblasts interspersed within the “outside” stroma. Note, RISH for *IGF1*, *CXCL13*, and *C7* was conducted on 7 additional BPH hubs (Supplemental Table 4).

By RNA in situ hybridization (RISH), we verified enriched expression of *IGF1* and *CXCL13* in the inner stroma adjacent to BPH hubs, including among hubs that were microdissected and transcriptionally profiled (exemplified by Hub-1 in Figure 3, A–D, and quantified in Supplemental Table 4), as well as in additional independent hubs (exemplified by Hub-7 in Figure 3, E–K). Importantly, enriched expression did not merely reflect an enrichment of prostate fibroblasts, since interstitial prostate fibroblasts — identified by expression of complement factor 7 (*C7*) (9) – were observed in both the inner and outer stroma (Figure 3, D, H, and K).

*IGF1* and *CXCL13* expression were not exclusive to the BPH hub–adjacent stroma. By examining the full prostate cross sections, we observed stromal *IGF1* and *CXCL13* expression elsewhere within BPH nodules, but always in association with adjacent epithelial hyperplasia and ductal branching (as observed for the BPH hubs) (examples in Supplemental Figure 1).

### IGF1 and CXCL13 are coexpressed in BPH fibroblasts and may function as paracrine factors.

**Figure 4:**
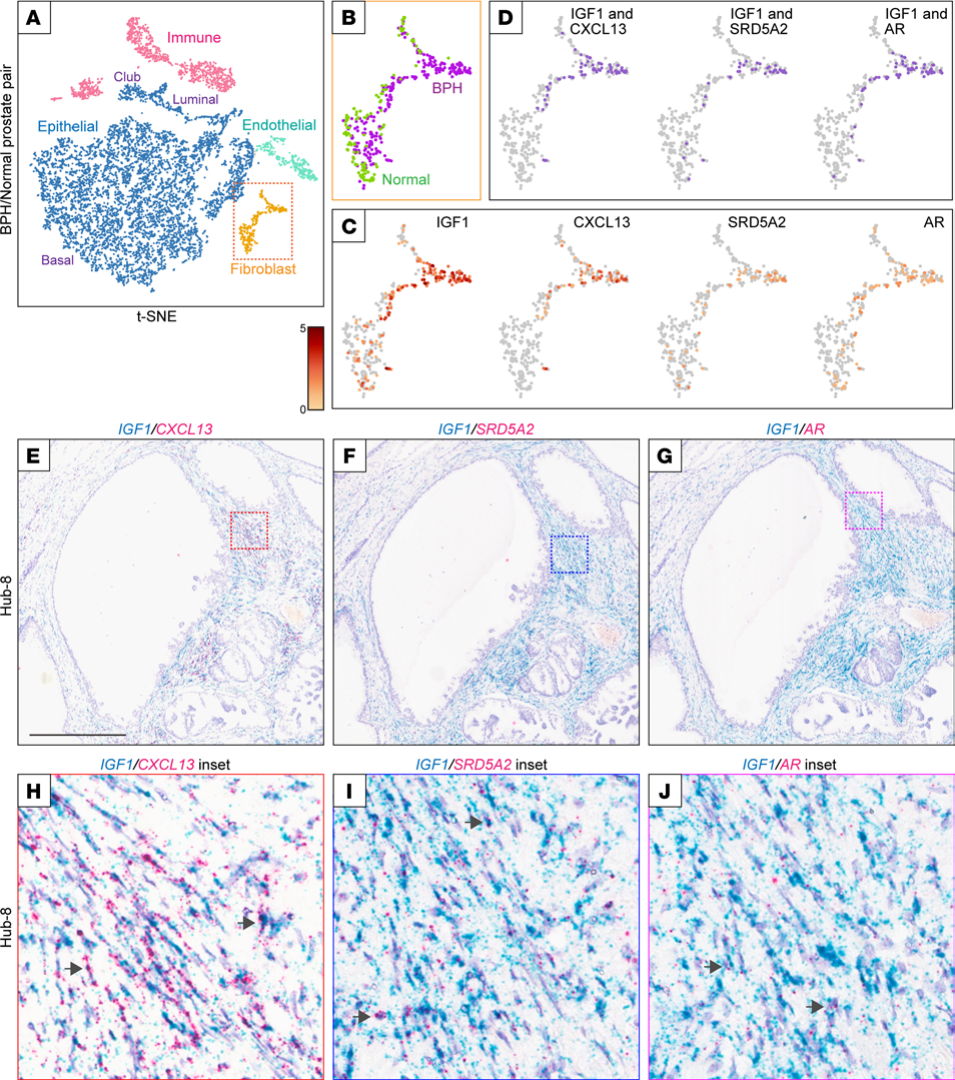
*IGF1* and *CXCL13* are coexpressed in BPH fibroblasts. (**A**–**D**) Single-cell RNA-Seq of paired BPH-normal human prostate tissue. (**A**) t-Distributed stochastic neighbor embedding (t-SNE) plot of combined BPH/normal prostate cells. Each dot represents an individual cell. Cell clusters (colored) are annotated by the expression of known cell type markers (e.g., *LUM* and *DCN* in fibroblasts). (**B**) Close-up of fibroblast cells contributed by BPH (purple) or normal prostate (green). (**C**) Fibroblast gene expression levels shown for *IGF1*, *CXCL13*, *SRD5A2*, and *AR*. Color bar depicts log_2_ transcript counts per cell. (**D**) Cell coexpression (purple) of *IGF1* with *CXCL13*, *SRD5A2*, or *AR*. (**E**–**J**) Two-color RNA in situ hybridization (RISH) of BPH tissue, exemplified for Hub-8, verifies cell coexpression of (**E** and **H**) *IGF1* (blue) with *CXCL13* (red); (**F** and **I**) *IGF1* (blue) with *SRD5A2* (red); and (**G** and **J**) *IGF1* (blue) with *AR* (red). Arrows identify representative cells with dual staining; scale bar is 500 μm, and all figure insets are an additional 8.7× magnification. Note, 2-color RISH for *IGF1*/*CXCL13*, *IGF1*/*SRD5A2*, and *IGF1*/*AR* was conducted on 1 additional BPH hub (Supplemental Table 4).

*IGF1* and *CXCL13* are both expressed in the inner stroma of BPH hubs. We next asked whether they were both expressed by the same cell. By single-cell RNA-Seq of paired BPH-normal human prostate tissue, we identified a discrete population of BPH fibroblasts (Figure 4, A and B, and Supplemental Figure 2). The BPH fibroblasts expressed both *IGF1* and *CXCL13*, as well as steroid 5 alpha-reductase 2 (*SRD5A2*) and androgen receptor (*AR*) (*P* ≤ 2.7 × 10^–5^, Hypergeometric test) (Figure 4, C and D). We verified coexpression of *IGF1* with *CXCL13*, *SRD5A2*, and *AR* by RISH (Figure 4, E–J, and quantified in Supplemental Table 4).

**Figure 5:**
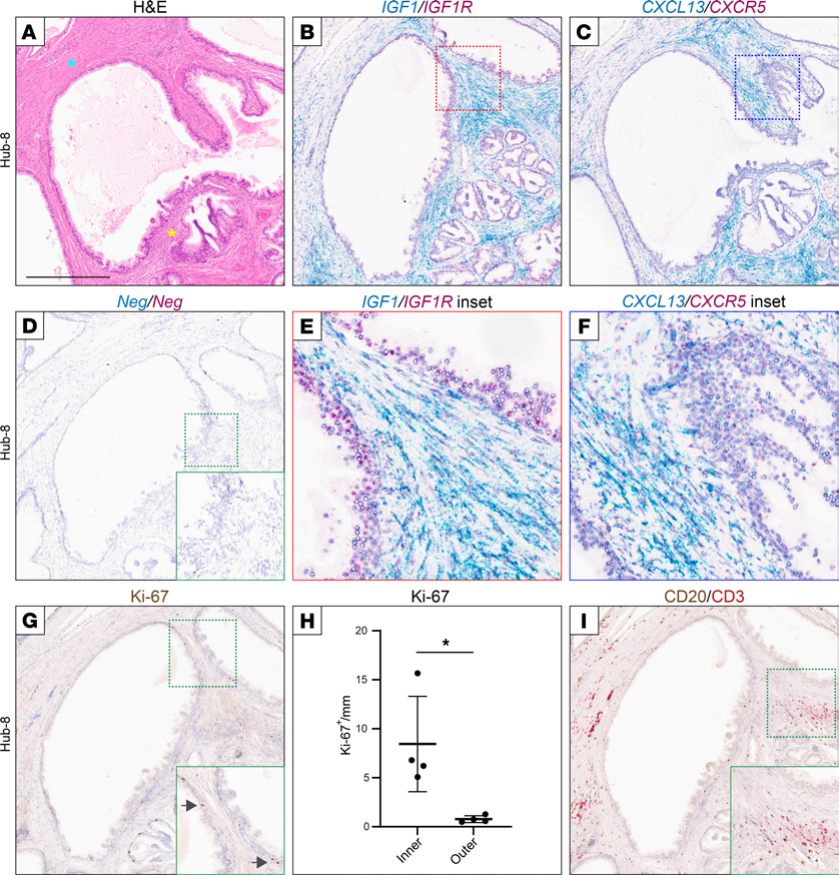
IGF1 and CXCL13 receptors are expressed in adjacent prostate ductal epithelium. (**A**) H&E stain of Hub-8. Inner stroma (yellow star) and outer stroma (blue star) are indicated; scale bar is 500 μm. (**B**–**F**) Two-color RNA in situ hybridization (RISH) of Hub-8 shown for (**B** and **E**) *IGF1* (blue) and *IGF1R* (red), (**C** and **F**) *CXCL13* (blue) and *CXCR5* (red), and (**D**) negative control probes. Note, 2-color RISH for *IGF1*/*IGF1R* and *CXCL13*/*CXCR5* was conducted on 3 additional BPH hubs (Supplemental Table 4). (**G**) IHC of Ki-67 identifies proliferating ductal epithelial cells adjacent to *IGF1*^+^ stroma. Arrows identify representative Ki-67^+^ cells. (**H**) Increased proliferating (Ki-67^+^) ductal epithelial cells on the side facing the inner/inductive stroma. Data from 4 hubs (see Supplemental Figure 2, quantified as Ki-67^+^ cells per millimeter duct; scale bar is 500 µm, and all figure insets are an additional 1.6× magnification. Mean ± SD shown. *P < 0.05; 2-sided paired Student’s *t* test. (**I**) Two-color IHC identifies B cells (CD20, brown) and T cells (CD3, red) within both the inner and outer stroma of hubs. Note, 2-color IHC for CD20/CD3 was conducted on 1 additional BPH hub (Hub-7).

Since IGF1 is expressed in hub-adjacent BPH stroma, we next asked whether its receptor — insulin-like growth factor 1 receptor (IGF1R) — was expressed in the hub ductal epithelium. Indeed, by 2-color RISH, we observed epithelial *IGF1R* expression adjacent to *IGF1*^+^ stroma (Figure 5, A, B, and E, and quantified in Supplemental Table 4). Perhaps more surprisingly (given the studied role of CXCL13 in lymphocyte chemoattraction), we also observed epithelial expression of the CXCL13 receptor — CXC chemokine receptor 5 (*CXCR5*) — adjacent to *CXCL13*^+^ stroma (Figure 5, C and F). Although expressed at lower levels than *IGF1R*, *CXCR5* expression was clearly visible in comparison with negative control probes (Figure 5D).

IGF1 is a growth factor known to promote cell proliferation (13). Consistent with that function, we observed by immunohistochemistry increased Ki-67^+^ (proliferating) epithelial cells in BPH hub epithelium adjacent to inner *IGF1*^+^*CXCL13*^+^ stroma, in comparison with the outer *IGF1*^–^*CXCL13*^–^ stroma (Figure 5, G and H, and Supplemental Figure 3) (*P* = 0.04, paired 2-sided Student’s *t* test). CXCL13 may have more than one function, but it has been best characterized as a lymphocyte chemoattractant (11). Perhaps surprisingly then, we observed B and T cell infiltrates within *both* the inner *CXCL13*^+^ stroma and the outer *CXCL13*^–^ stroma (example shown in Figure 5I).

### IGF1 but not CXCL13 is expressed in human embryonic prostate stroma.

**Figure 6:**
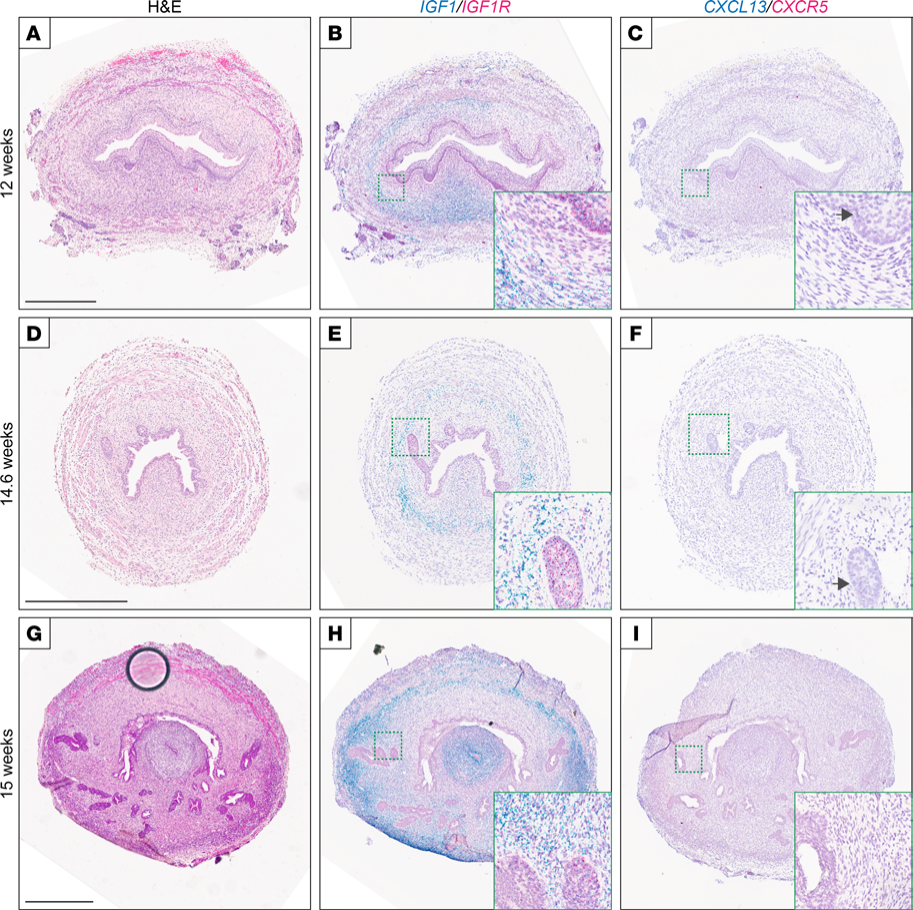
*IGF1* but not *CXCL13* is expressed in stroma of human fetal prostates undergoing branching morphogenesis. Shown are cross sections of human fetal prostates at gestational ages (**A**–**C**) 12 weeks showing the urethra, (**D**–**F**) 14.6 weeks showing ductal budding from the urethra, and (**G**–**I**) 15 weeks showing ductal branching. (**A**, **D**, and **G**) H&E stains; scale bar is 500 μm. Note, the large black circle in **G** is a bubble under the coverslip. (**B**, **E**, and **H**) Two-color RNA in situ hybridization (RISH) of *IGF1* (blue) and *IGF1R* (red). (**C**, **F**, and **I**) Two-color RISH of *CXCL13* (blue) and *CXCR5* (red). Arrows mark occasional epithelial cells with low-intensity *CXCL13* and/or *CXCR5* expression; scale bar is 500 µm, and all figure insets are an additional 3.2×–4.5× magnification.

McNeal’s hypothesis — that BPH represents a reawakening of embryonic patterns of stromal induction — would imply that *IGF1* and *CXCL13* are also expressed in human embryonic prostatic stroma. To investigate this possibility, we carried out RISH on human fetal prostate tissues (obtained from elective terminations) of 12–15 weeks of gestation, within the time frame of embryonic ductal branching (14). We observed marked *IGF1* expression within the embryonic prostate stroma encircling the urethra (Figure 6, A and B), and adjacent to new ductal budding (Figure 6, D and E) and ductal branching (Figure 6, G and H), as well as *IGF1R* expression in the prostatic epithelium (Figure 6, B, E, and H). In contrast, *CXCL1*3 expression was not apparent in the embryonic prostate stroma, although low-level expression of *CXCL13* and/or *CXCR5* could be observed in a small minority of epithelial cells (Figure 6, C, F, and I).

### Inhibition of IGF1 signaling abolishes BPH spheroid and organoid formation.

**Figure 7:**
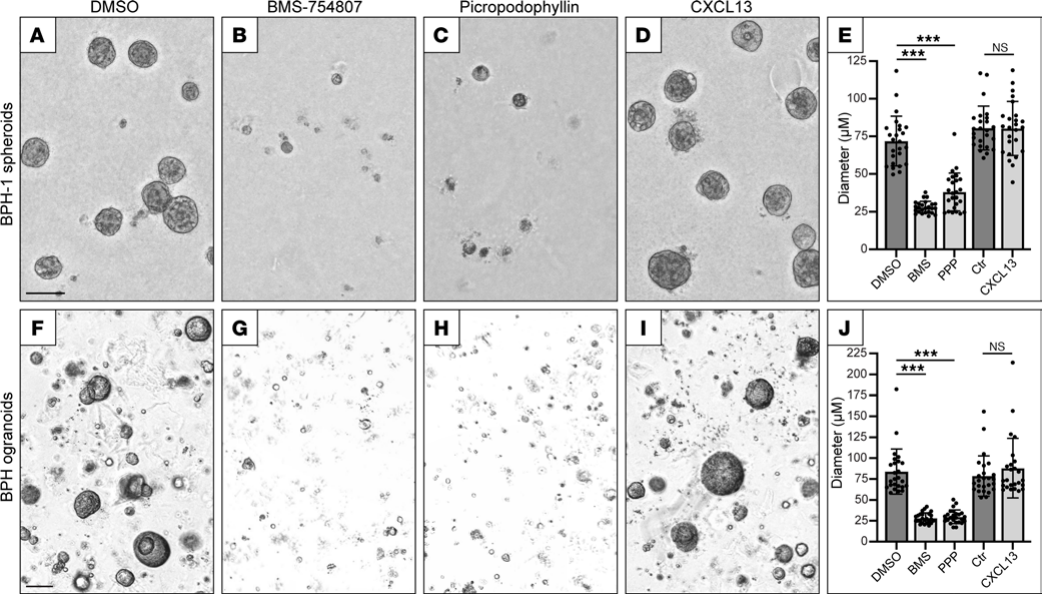
Inhibition of IGF1 signaling abolishes BPH spheroid and organoid formation. (**A**–**E**) BPH-1 cell spheroids grown in Matrigel to 10 days and (**F**–**J**) patient-derived BPH organoids grown in Matrigel to 14 days, in media containing (**A** and **F**) vehicle (DMSO), (**B** and **G**) IGF1R inhibitor BMS-754807 (1 μM), (**C** and **H**) IGF1R inhibitor picropodophyllin (PPP) (1 μM), or (**D** and **I**) recombinant human CXCL13 (100 ng/mL). Scale bar is 100 μm. (**E**) BPH-1 spheroid and (**J**) BPH organoid generation quantified as the largest 25 diameters (μM) in a random 10× microscope field. Mean ± SD shown. ***P < 0.001; 2-sided Student’s *t* test, with Bonferroni’s correction applied for multiple comparisons. Each experiment was conducted twice. “Ctr” is media-alone comparison control for CXCL13.

Our spatial transcriptomics identified stromal IGF1 as a candidate inducer of BPH ductal epithelial hyperplasia, proliferation (Ki-67 staining), and/or neoductal growth. To evaluate IGF1 function on glandular epithelium, we used 2 different 3D cell culture models: spheroid culture of BPH-1 cells (a prostate epithelial cell line established from human BPH tissue) (15) and organoid culture (16) of primary patient-derived BPH epithelial cells. Since IGF1 (and/or the cross-activating insulin) (17) is a component of BPH-1 cell culture media (containing fetal bovine serum), prostate organoid media (containing insulin), and Matrigel (where growth factor–depleted Matrigel retains IGF1), we evaluated the effect of IGF1R inhibition. Two small molecular IGF1R tyrosine kinase inhibitors — BMS-754807 (18) and picropodophyllin (PPP) (19) — at 1 μM thwarted BPH-1 spheroid formation and BPH organoid formation in Matrigel (*P* < 0.001; 2-sided Student’s *t* test) (Figure 7, A–C, E–H, and J, and Supplemental Figure 4).

Given the observed *CXCR5* expression in BPH epithelium, we also sought to evaluate CXCL13 function on BPH epithelium. Since CXCL13 is not a known component of BPH-1 cell media or prostate organoid media, we evaluated the effect of CXCL13 addition on BPH-1 spheroid and BPH organoid culture. Physiologic concentrations of recombinant human CXCL13 (100 ng/mL) had no discernable impact on spheroid or organoid formation (Figure 7, D, E, I, and J). However, we note that expression of the CXCL13 receptor *CXCR5*, by quantitative reverse transcription PCR (qRT-PCR), was substantially lower compared with *IGF1R* in BPH-1 spheroids and BPH organoids (Supplemental Figure 5).

## Discussion

Decades ago, McNeal observed that the tangent ducts bordering BPH nodules display epithelial hyperplasia and neoductal growth exclusively on the side facing into the nodule, suggesting a response to inductive stroma (3). Here, by spatial transcriptomics of the inner versus outer stroma neighboring these BPH hubs, we have identified candidate stromal inductive factors, including IGF1 and CXCL13.

From mouse knockout studies, IGF1 was previously linked to prostate development and ductal branching morphogenesis (10), and indeed IGF signaling has long been implicated in BPH (20). However, much of the evidence has been circumstantial, and uncertainty has remained even over which prostate cell type(s) produce (or respond) to IGFs. In one study, primary human BPH fibroblasts were found to express *IGF2* but not *IGF1* (21). In another study, some but not other primary human prostate fibroblast cultures (albeit none derived from BPH tissue) showed androgen-stimulated expression of *IGF1* (22). Other studies have implicated *IGF1* expression from prostate epithelial cells and even macrophages (23). Thus, importantly, we believe our study is the first to identify high *IGF1* expression in human BPH fibroblasts *at the site* of *IGF1R*^+^ epithelial hyperplasia and neoductal growth, providing “smoking gun” evidence of the key role of stromal IGF1 in BPH nodular morphogenesis.

Our finding of high stromal *IGF1* expression adjacent to BPH hubs is notable, but equally striking is what we did not find. Other growth factors — including FGFs, TGFs, BMPs, WNTs, and SHH — also have reported roles in fetal/neonatal prostate development (5, 6) and by extension have been implicated in BPH (1, 7). Yet among those growth factors, our studies identified only *IGF1* (and *IGF2*) as top enriched genes. Our findings singularly highlight IGF1 as a critical growth factor in BPH nodular proliferation and therefore as a persuasive target for therapy. Our results may also explain why hyperinsulinemia is a risk factor for BPH (24); high insulin levels can crossactivate the IGF1R (17). Nonetheless, other growth factors — including bone morphogenetic protein 5 (BMP5) that we previously found highly overexpressed in bulk BPH tissue (8) — likely contribute to BPH, albeit exhibiting less polarized expression in the inner stroma, or perhaps with activity at earlier stages of nodular growth.

We recently reported markedly elevated expression of *CXCL13* in bulk BPH tissue, where it was also part of a 65-gene BPH stromal signature that correlated with BPH symptom severity (8), but it had until then never been implicated in BPH. CXCL13 has been characterized as a lymphocyte chemoattractant (11). Nonetheless, in our preliminary studies, we observed lymphocyte infiltrates within both the inner and outer stroma. Notably, we observed expression of *CXCR5* (the only known CXCL13 receptor) in BPH epithelium. This finding suggests the possibility that CXCL13 functions as a paracrine factor in the growth or some other characteristic of adjacent prostatic epithelium. While our studies in 3D culture did not reveal that function, future studies (including in vivo) should inform the function(s) of CXCL13 in BPH pathogenesis.

The top gene transcripts overexpressed in inner (presumptive inductive) stroma were enriched for secreted factors, as predicted by McNeal’s hypothesis. Notably, their known functions suggest roles not only in epithelial induction (*IGF1*, *IGF2*), but also in the proliferation, differentiation, or function of other cell types, including immune cells (*CXCL13*) (11), endothelial cells (*PROK1*) (25), and peripheral neurons (*EDN3*) (26). This finding underscores that BPH nodular proliferation is more than merely epithelial and fibroblast proliferation, but rather encompasses the morphogenesis of diverse new tissue elements. The function of other top-ranked inner stroma gene transcripts (e.g., *PTN*, *PLAC9*) remains to be investigated. Future studies will also evaluate the protein expression of the above factors (which is notoriously challenging for secreted proteins). Also of future interest are the gene transcripts enriched within the outer stroma of hubs, which might include factors that constrain epithelial proliferation. Single-cell spatial approaches will also refine the list and cell type source of inductive factors, though we note that current approaches would have missed *CXCL13*, which is not included in existing catalog gene sets.

Our studies were motivated by McNeal’s observations and hypothesis that BPH is the reawakening of embryonic inductive activity by adult prostate stroma. Though mesenchymal induction of prostatic epithelial development was elucidated in the mouse and rat, recent studies demonstrated that *human* urogenital sinus mesenchyme is also an inducer of prostatic epithelial development (27). It is therefore notable that our findings only partly support McNeal’s hypothesis. *IGF1* is expressed in BPH inner (inductive) stroma and in the stroma of human fetal prostates undergoing ductal branching morphogenesis. However, *CXCL13* expression is absent from the stroma of those fetal prostates. Thus, in the most straightforward interpretation, BPH nodular proliferation is not simply the reactivation of embryonic inductive patterns. Future studies that employ single-cell profiling of active chromatin (e.g., by ATAC-seq) (28) may reveal the processes regulating *IGF1* and *CXCL13* expression in BPH fibroblasts, and whether these reflect normal embryonic patterns or focal responses, for example to hormonal changes, inflammation, or aging.

Our studies also have several therapeutic implications. Most notably, 2 small molecule IGF1R inhibitors, each with different off-target profiles (18, 19), abolished BPH-1 spheroid and patient-derived BPH organoid growth in cell culture. While follow-up studies (including in vivo studies) are needed, our findings suggest the potential efficacy of IGF1R inhibitors as a disease-targeted approach in preventing or managing BPH and its associated lower urinary tract symptoms. In cancer clinical trials, IGF1R inhibitors have shown manageable toxicity (albeit with limited clinical benefit against many cancers) (29); thus, they may find utility in BPH, particularly if delivery or activity can be restricted to the prostate. Indeed, inhibition of mTOR (which functions downstream of IGF1R) was reported to reduce prostate size (30). And saw palmetto extract, an alternative BPH therapy, may modulate the IGF1 signaling pathway (31).

Also intriguing is that the BPH fibroblasts expressing *IGF1* and *CXCL13* also express *SRD5A2* (5α-reductase) and *AR*. Currently, 5α-reductase inhibitors (e.g., finasteride and dutasteride) are a first-line therapy for BPH, where they inhibit the conversion of testosterone to the more potent dihydrotestosterone, leading to the involution of androgen-dependent prostatic epithelium and prostate shrinkage (26). Of note, during prostate development, androgens act primarily on embryonic mesenchyme to prompt the secretion of paracrine-acting epithelial growth factors (32). Thus, it will be of interest to determine whether 5α-reductase inhibitors also affect the AR^+^ BPH fibroblast expression of *IGF1*, *CXCL13*, and/or other secreted/inductive factors. If so, then these therapies might prove “more targeted” to BPH disease mechanisms than currently recognized.

## Methods

### Prostate tissue specimens.

Human BPH tissue specimens were obtained from radical prostatectomy cases (done for prostate cancer) at the Stanford Hospital, with IRB approval and patient informed consent. LCM studies were done using archived FFPE blocks, while single-cell RNA-Seq and patient-derived organoid studies used fresh BPH tissue. Patient age, prostate size, and International Prostate Symptom Score are listed in Supplemental Table 1. Human fetal prostate specimens were obtained from elective terminations done at UCSF, with IRB approval and patient informed consent.

### LCM and RNA-Seq.

For each BPH hub, LCM was done on 3 consecutive 10 μM FFPE sections, using an Arcturus XT System. RNA was isolated from captured tissue using QIAGEN RNeasy FFPE kit, then quantified by Agilent 2100 BioAnalyzer. RNA-Seq libraries were constructed from 2 ng RNA input using Takara SMARTer Stranded Total RNA-seq kit v3 — Pico Input Mammalian. RNA-Seq libraries were evaluated by Agilent BioAnalyzer, then sequenced on an Illumina NovaSeq 6000 (Novogene) to an average 46 million read pairs per sample.

### RNA-Seq data analysis.

Illumina sequence reads were used to quantify transcript abundance as transcripts per million (TPM) using kallisto (33). TPM data were processed sequentially by thresholding low values (to 0.01), log_2_ transforming, mean centering genes and samples, and excluding genes with low variability in expression (SD < 1). Differentially expressed genes (inner/inductive versus outer stroma) were identified using paired-SAM (34), which estimates false discovery rates against randomly permutated samples. Pathway analysis was done using IPA (QIAGEN). Predicted secreted proteins were identified from the Human Protein Atlas (12).

### RISH and immunohistochemistry.

RISH was done by manual RNAscope Chromogenic assays, using RNAscope 2.5 HD Assay—BROWN or Duplex Assay according to the manufacturer (ACDBio). Chromogenic probes were Hs-IGF1, Hs-CXCL13, Hs-C7, Hs-CXCL13-C2, Hs-IGF1R-C2, Hs-SRD5A2-C2, and Hs-AR-C2. Semiquantitative scoring of RNAscope assays (based on dots per cell, 0–4 scale) was done per ACDBio guidelines. Immunohistochemistry was done using Tris-EDTA antigen retrieval, with ImmPRESS HRP Detection Kit and ImmPRESS Duet Double Staining Polymer Kit (HRP/AP) (Vector Laboratories). Primary antibodies were anti–Ki-67 (D2H10, 1:400) (Cell Signaling Technology), anti-CD20 (L26, 1:400) (MilliporeSigma), and anti-CD3 (MRQ-39, 1:800) (MilliporeSigma).

### Single-cell RNA-Seq.

For single-cell RNA-Seq, fresh human BPH and normal prostate tissues were separately disaggregated to single cells by sequential incubation with collagenase/hyaluronidase, trypsin, and dispase, following Prostate Tissue Dissociation protocol (STEMCELL Technologies) (35). Red blood cells were lysed by Ammonium Chloride Solution (STEMCELL Technologies), and then live cells (propidium iodide negative) were flow-sorted (Stanford Shared FACS Facility). Single-cell RNA-Seq libraries were generated separately for BPH and normal prostate tissue using 10x Genomics Chromium Controller (Stanford Functional Genomics Facility), reagent kits, and protocols. Libraries were sequenced on an Illumina X Ten (Novogene). Single-cell data were processed using Cell Ranger, and BPH and normal prostate data were then aggregated (cellranger aggr command) and visualized using Loup Browser (10x Genomics).

### Prostate cell culture experiments.

BPH-1 cells (15) were obtained from the DSMZ cell culture repository, then verified by short tandem repeat profiling (ATCC). For spheroid assays, 50,000 cells (per 6-well plate well) were plated in growth factor–reduced Matrigel (Corning) using Matrigel “3D on-top” assays (36). BPH-1 cell media comprised RPMI-1640 media with 5% FBS. For patient-derived organoids, fresh human BPH tissue was disaggregated to single cells as detailed above. Cells were then plated in Matrigel as droplets (20,000–50,000 cells) (16) or “3D on-top” assays (100,000–200,000 cells) in a 24-well plate (36). BPH organoids were grown in Clevers media, exactly as described (16), with media changes every 2–3 days. BMS-754807 and PPP were obtained from SelleckChem and human recombinant CXCL13 from PeproTech. Spheroids and organoids were imaged using a Keyence BZ-X810 and diameters measured using ImageJ (NIH). In some experiments, spheroids were harvested from Matrigel by dilution in cold PBS with 5 mM EDTA, then fixed in 4% paraformaldehyde, and embedded in paraffin for subsequent sectioning and H&E staining. For qRT-PCR, spheroids and organoids were harvested as above, and then RNA was isolated using QIAGEN RNeasy Mini Kit. qRT-PCR was done using TaqMan Gene Expression Assays, with probe sets for *IGF1R* (Hs00609566_m1), *CXCR5* (Hs00540548_s1), and *GAPDH* Endogenous control (Applied Biosystems), with TaqMan Fast Advanced Master Mix (Applied Biosystems), on a QuantStudio 5 Real-Time PCR System (Thermo Fisher Scientific).

### Statistics.

Unless otherwise indicated, a 2-sided Student’s *t* test was performed to assess statistical significance, using GraphPad Prism 10.0, where *P* < 0.05 was considered significant. Paired 2-class analysis of RNA-Seq data was done using SAM (34), which estimates false discovery rates against randomly permutated samples. Pathway analysis was done using IPA, where *P* values are calculated by Hypergeometric test.

### Study approval.

The study was approved by the Stanford University IRB. Written informed consent was received from participants prior to inclusion in the study.

### Data availability.

All RNA-Seq and single-cell RNA-Seq data are available from the NIH Database of Genotypes and Phenotypes (phs003477.v1.p1). Supporting Data Values associated with the manuscript figures are available as supplemental material, in the Excel file.

## Author contributions

ASP, CAK, GC, LSB, RBW, JDB, and JRP conceived and planned the studies; ASP, ZS, SV, and JRP performed experiments; GRC and LSB contributed key resources; ASP, NB, and JRP analyzed data; and ASP and JRP wrote the manuscript.

## Supplementary Material

Supplemental data

Supporting data values

## Version 1 11/16/2023

In-Press Preview

## Version 2 01/23/2024

Electronic publication
