# Supplementary material for: Spatial transcriptomics identifies candidate stromal drivers of benign prostatic hyperplasia
Source: JCI Insight. 2024 Jan 23;9(2):e176479. doi: 10.1172/jci.insight.176479 (PMC10906230; doi:10.1172/jci.insight.176479)
Supplement: Supplemental data [file jciinsight-9-176479-s163.pdf]

Spatial transcriptomics identifies candidate stromal drivers of benign prostatic hyperplasia

Anna S. Pollack[1], Christian A. Kunder[1], Noah Brazer[1], Zhewei Shen[1], Sushama Varma[1], Robert B. West[1], Gerald R. Cunha[2], Laurence S. Baskin[2], James D. Brooks[3], Jonathan R. Pollack[1,*]

Departments of [1]Pathology, and [3]Urology, Stanford University School of Medicine, 300 Pasteur Drive, Stanford, California, 94305; [2]Department of Urology, University of California at San Francisco, San Francisco, California, 94143

Supplementary Table 1. Human prostate tissue samples

Supplementary Table 2. Top differentially expressed genes

Supplementary Table 3. Top enrichment by Ingenuity Pathway Analysis

Supplemental Table 4. RNA in situ hybridization assays and quantification

Supplementary Figure 1. *IGF1* and *CXCL13* expression is not exclusive to BPH hub-adjacent stroma.

Supplemental Figure 2. Single-cell RNAseq of prostate identifies BPH fibroblast cluster

Supplementary Figure 3. Quantifying Ki-67+ ductal cells facing inner versus outer stroma.

Supplementary Figure 4. Inhibition of IGF1 signaling thwarts BPH-1 spheroid formation.

Supplementary Figure 5. *CXCR5* is expressed in BPH spheroids and organoids at substantially lower levels than *IGF1R*.

Supplementary Table 1. Human prostate tissue samples

Sample ID	Source	Patient age (yrs)	Prostate size (cc)	International Prostate Symptom Score (IPSS)[a]
Hub-1, Hub-2	Radical prostatectomy	73	28	4
Hub-3	Radical prostatectomy	70	66	na
Hub-4	Radical prostatectomy	79	32	15
Hub-5	Radical prostatectomy	59	56	9
Hub-6	Radical prostatectomy	64	65	6
Hub-7	Radical prostatectomy	67	35	22
Hub-8	Radical prostatectomy	69	38	14
Hub-9	Radical prostatectomy	73	45	na
scRNAseq	Radical prostatectomy	68	65	18
Organoid	Cysto-prostatectomy	73	na	na
Fetal-12 weeks	Elective termination			
Fetal-14.6 weeks	Elective termination			
Fetal-15 weeks	Elective termination			

[a]Mild (0-7); Moderate (8-19); Severe (20-35)

Supplementary Table 2. Top differentially-expressed genes

Up in Inner	Gene ID[a]	Alternate Gene ID	Gene Name	T-statistic value	Fold Change	False Discovery Rate (%)
	CXCL13		C-X-C Motif Chemokine Ligand 13	5.62	7.08	0.00
	IGF1		Insulin Like Growth Factor 1	3.88	5.65	6.66
	EDN3		Endothelin 3	3.66	4.25	6.66
	CFD		Complement Factor D	3.65	3.48	6.66
	PPP1R1B		Protein Phosphatase 1 Regulatory Inhibitor Subunit 1B	3.54	4.24	6.66
	PTGDS		Prostaglandin D2 Synthase	3.16	2.68	11.75
	IGF2		Insulin Like Growth Factor 2	3.07	3.41	11.75
	PROK1		Prokineticin 1	2.91	3.77	11.75
	PTN		Pleiotrophin	2.89	4.00	11.75
	LYPLAL1		Lysophospholipase Like 1	2.87	1.81	11.75
	GOLGA8M		Golgin A8 Family Member M	2.85	2.51	11.75
	PLAC9		Placenta Associated 9	2.83	2.35	11.75
	CAPN6		Calpain 6	2.75	2.47	17.95
	AFF2		ALF Transcription Elongation Factor 2	2.68	2.91	17.95
	LRRC37A6P		Leucine Rich Repeat Containing 37 Member A6, Pseudogene	2.66	1.90	17.95
	MTND4P16		MT-ND4 Pseudogene 16	2.62	3.79	17.95
	TMEM132C		Transmembrane Protein 132C	2.61	3.73	17.95
	RPE65		Retinoid Isomerohydrolase RPE65	2.60	2.74	17.95
	NELL2		Neural EGFL Like 2	2.59	3.47	17.95
	PENK		Proenkephalin	2.57	4.11	17.95
	GDPD3		Glycerophosphodiester Phosphodiesterase Domain Containing 3	2.56	1.77	17.95
	RTN4RL2		Reticulon 4 Receptor Like 2	2.56	2.56	17.95
	HS6ST2		Heparan Sulfate 6-O-Sulfotransferase 2	2.54	3.58	17.95
	SLC15A2		Solute Carrier Family 15 Member 2	2.53	2.10	17.95
	SLC38A5		Solute Carrier Family 38 Member 5	2.53	2.57	17.95
	MARCKSL1		MARCKS Like 1	2.53	1.81	17.95
	LRRC17		Leucine Rich Repeat Containing 17	2.53	3.36	17.95
	C1QL1		Complement C1q Like 1	2.52	5.73	17.95
	MFAP2		Microfibril Associated Protein 2	2.49	1.73	19.54
	FAM13C		Family With Sequence Similarity 13 Member C	2.44	3.00	19.54
	DDX18P1		DEAD-Box Helicase 18 Pseudogene 1	2.44	2.34	24.97
	PNMT		Phenylethanolamine N-Methyltransferase	2.39	2.53	24.97
	TNFSF13		TNF Superfamily Member 13	2.39	2.11	24.97
	LINC00693		Long Intergenic Non-Protein Coding RNA 693	2.35	1.70	24.97
	ENPP7P10		Ectonucleotide Pyrophosphatase/Phosphodiesterase 7 Pseudogene 10	2.33	2.60	28.69
	PPIF		Peptidylprolyl Isomerase F	2.32	2.65	28.69
	TMEM100		Transmembrane Protein 100	2.31	3.34	28.69
	KCP		Kielin Cysteine Rich BMP Regulator	2.31	2.05	28.69
	SMOC1		SPARC Related Modular Calcium Binding 1	2.31	2.39	28.69
	NSUN7		NOP2/Sun RNA Methyltransferase Family Member 7	2.31	2.18	28.69
**Up in Outer**	**Gene ID[a]**	**Alternate Gene ID**	**Gene Name**	**T-statistic value**	**Fold Change**	**False Discovery Rate (%)**
	EFNA5		Ephrin A5	-4.01	0.31	0.00
	ID1		Inhibitor Of DNA Binding 1	-3.79	0.32	0.00
	BGN		Biglycan	-3.72	0.31	0.00
	MEGF6		Multiple EGF Like Domains 6	-3.69	0.26	0.00
	ACHE		Acetylcholinesterase (Cartwright Blood Group)	-3.66	0.29	0.00
	C4A		Complement C4A (Rodgers Blood Group)	-3.53	0.32	0.00
	A2M		Alpha-2-Macroglobulin	-3.49	0.34	0.00
	NXPH3		Neurexophilin 3	-3.40	0.31	0.00
	MMP7		Matrix Metallopeptidase 7	-3.38	0.35	0.00
	KLF2		KLF Transcription Factor 2	-3.35	0.20	0.00
	ASB5		Ankyrin Repeat And SOCS Box Containing 5	-3.17	0.17	5.87
	DUSP1		Dual Specificity Phosphatase 1	-3.16	0.29	5.87
	CCN1	CYR61	Cellular Communication Network Factor 1	-3.09	0.17	5.87
	ITGA8		Integrin Subunit Alpha 8	-3.09	0.28	5.87
	CCN2	CTGF	Cellular Communication Network Factor 2	-3.03	0.22	6.81
	MOXD1		Monooxygenase DBH Like 1	-3.02	0.13	6.81
	GCA		Grancalcin	-2.99	0.40	6.81
	GCHFR		GTP Cyclohydrolase I Feedback Regulator	-2.94	0.26	8.68
	DUSP5		Dual Specificity Phosphatase 5	-2.85	0.24	8.68
	CCDC3		Coiled-Coil Domain Containing 3	-2.83	0.27	8.68
	THBS2		Thrombospondin 2	-2.76	0.24	11.10
	ITGBL1		Integrin Subunit Beta Like 1	-2.75	0.14	11.10
	PDZD4		PDZ Domain Containing 4	-2.71	0.22	11.10
	FOS		Fos Proto-Oncogene, AP-1 Transcription Factor Subunit	-2.67	0.15	11.75
	CCN3	NOV	Cellular Communication Network Factor 3	-2.65	0.19	11.75
	LBH		LBH Regulator Of WNT Signaling Pathway	-2.63	0.40	11.75
	PCDH17		Protocadherin 17	-2.63	0.24	11.75
	PERP		P53 Apoptosis Effector Related To PMP2	-2.58	0.37	14.46
	NR4A1		Nuclear Receptor Subfamily 4 Group A Member 1	-2.56	0.26	14.46
	ADNP2		ADNP Homeobox 2	-2.55	0.35	14.46
	NCF1C		Neutrophil Cytosolic Factor 1C Pseudogene	-2.55	0.45	14.46
	CPXM2		Carboxypeptidase X, M14 Family Member 2	-2.52	0.37	14.46
	ATF3		Activating Transcription Factor 3	-2.50	0.22	14.46
	HGF		Hepatocyte Growth Factor	-2.49	0.40	17.95
	BEX2		Brain Expressed X-Linked 2	-2.46	0.35	17.95
	LTBP2		Latent Transforming Growth Factor Beta Binding Protein 2	-2.45	0.40	17.95
	FOSB		FosB Proto-Oncogene, AP-1 Transcription Factor Subunit	-2.41	0.30	17.95
	PTGS2		Prostaglandin-Endoperoxide Synthase 2	-2.39	0.43	19.54
	GGACT		Gamma-Glutamylamine Cyclotransferase	-2.36	0.38	19.54
	RASD1		Ras Related Dexamethasone Induced 1	-2.36	0.44	19.54

[a] Red text indicates predicted secreted protein

Supplementary Table 3. Top enrichment by Ingenuity Pathway Analysis		
**Enriched in Inner (top 200 genes)**	**Molecular and Cellular Functions**	**P-value range**
	Cellular Development	1.03E-03 - 4.87E-07
	Cellular Movement	1.03E-03 - 1.89E-06
	Lipid Metabolism	1.12E-03 - 2.44E-06
	Molecular Transport	1.11E-03 - 2.44E-06
	Small Molecule Biochemistry	1.12E-03 - 2.44E-06
	**Physiological System Development and Function**	**P-value range**
	Connective Tissue Development and Function	1.11E-03 - 4.87E-07
	Tissue Development	1.03E-03 - 4.87E-07
	Tissue Morphology	1.11E-03 - 5.50E-07
	Organ Morphology	1.01E-03 - 3.61E-06
	Reproductive System Development and Function	1.07E-03 - 3.61E-06
**Enriched in Outer (top 200 genes)**	**Molecular and Cellular Functions**	**P-value range**
	Cellular Development	1.13E-05 - 5.15E-18
	Cellular Movement	9.30E-06 - 2.25E-17
	Cellular Growth and Proliferation	1.13E-05 - 6.16E-12
	Gene Expression	4.59E-08 - 2.04E-11
	Cell Death and Survival	9.84E-06 - 4.75E-11
	**Physiological System Development and Function**	**P-value range**
	Connective Tissue Development and Function	1.13E-05 - 5.15E-18
	Tissue Development	1.13E-05 - 5.15E-18
	Organismal Development	1.15E-05 - 2.34E-13
	Skeletal and Muscular System Development and Function	1.21E-05 - 3.28E-13
	Embryonic Development	1.11E-05 - 4.76E-13

Supplemental Table 4. RNA in situ hybridization assays and quantification[a]

**Sample ID**	**Inner *IGF1***	**Outer *IGF1***	**Inner *CXCL13***	**Outer *CXCL13***	**Inner *C7***	**Outer *C7***	***SRD5A2***	***AR***	***IGF1R* (epithelium)**	***CXCR5* (epithelium)**
Hub-1	4	1	3	0	4	4	na	na	3	1
Hub-2	4	1	3	0	4	4	na	na	na	na
Hub-3	4	1	2	0	4	4	na	na	na	na
Hub-4	3	1	2	0	na	na	na	na	na	na
Hub-5	4	0	2	0	4	4	na	na	na	na
Hub-6	4	1	3	0	4	4	na	na	na	na
Hub-7	4	1	3	0	4	4	na	na	3	2
Hub-8	4	1	4	1	4	4	3	2	4	2
Hub-9	4	2	3	0	4	4	3	2	3	2
**P-value[b]**	<0.001		<0.001							

[a]Semi-quantitative scoring of predominant pattern, per ACDBio guidelines:

<u>Score</u>	<u>Criteria</u>
0	No staining or <1 dot/ 10 cells
1	1-3 dots/cell
2	4-9 dots/cell. None or very few dot clusters
3	10-15 dots/cell and <10% dots are in clusters
4	>15 dots/cell and >10% dots are in clusters

[b]Inner versus Outer by paired 2-sided Student's T-test

Supplemental Figure 1A

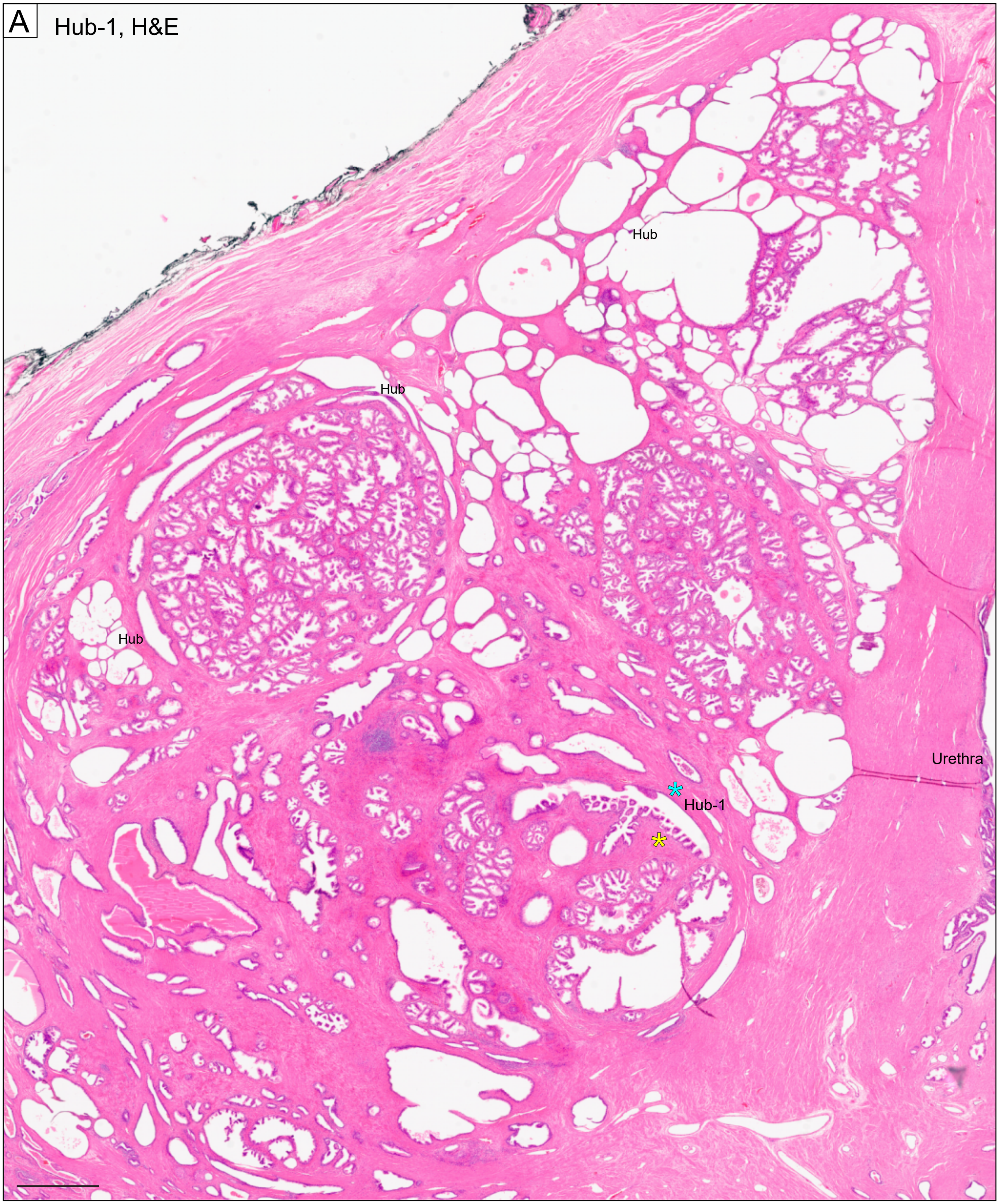

Supplemental Figure 1. *IGF1* and *CXCL13* expression is not exclusive to BPH hub-adjacent stroma. (A-C) Cross-section of transition zone containing multiple BPH nodules. Scale bar is 1mm. **(A)** H&E stain. **(B)** *IGF1* expression by RISH (brown staining). **(C)** *CXCL13* expression by RISH (brown staining). Location of Hub-1 plus other hubs is indicated. Representative foci of *IGF1*+/*CXCL13*+ stroma are highlighted by yellow stars, and foci of *IGF1*-/*CXCL13*- stroma by blue stars. Note that *IGF1*+/*CXCL13*+ stroma is observed elsewhere within BPH nodules, but always in close proximity to epithelial hyperplasia and ductal branching.

Supplemental Figure 1B

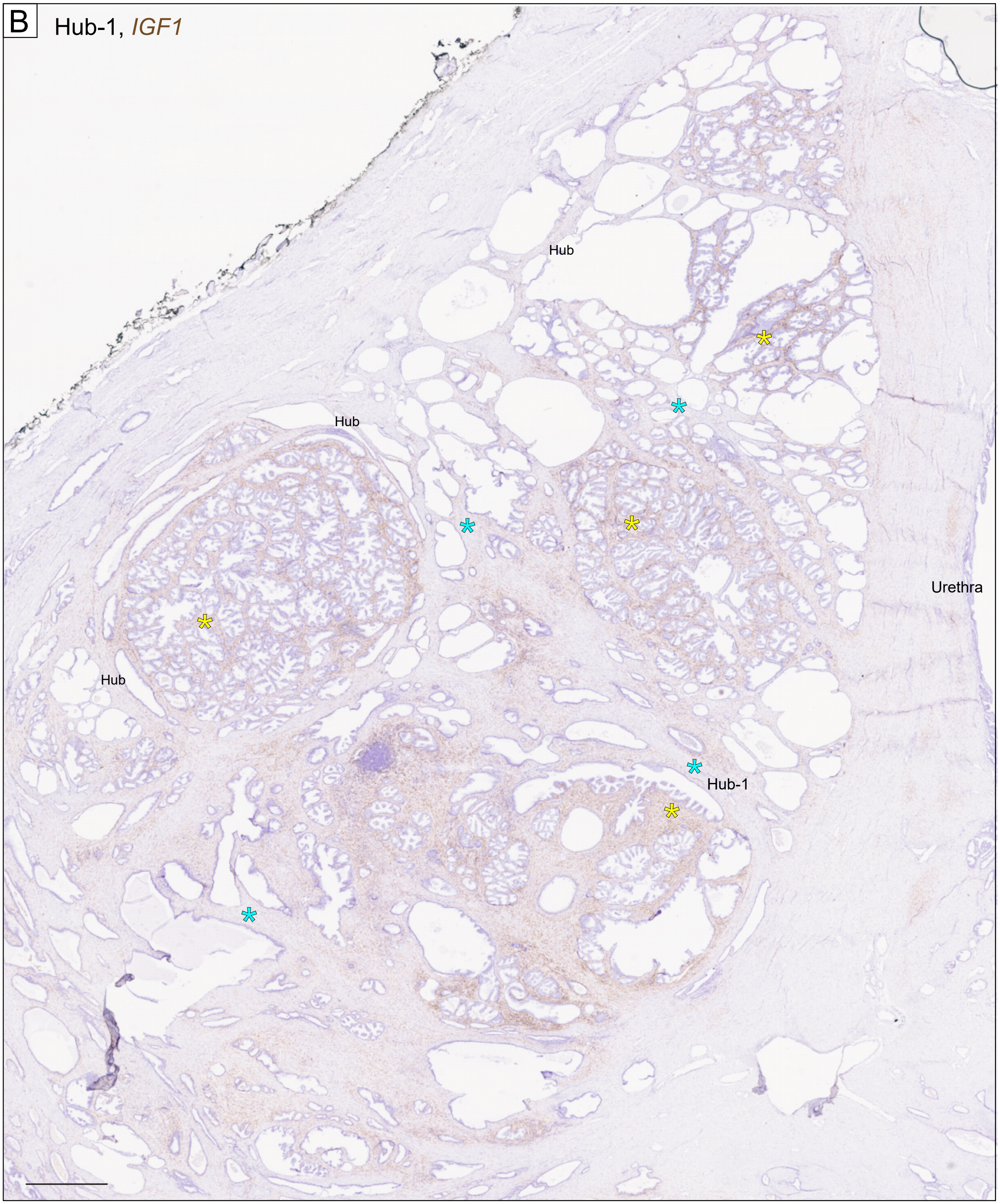

Supplemental Figure 1C

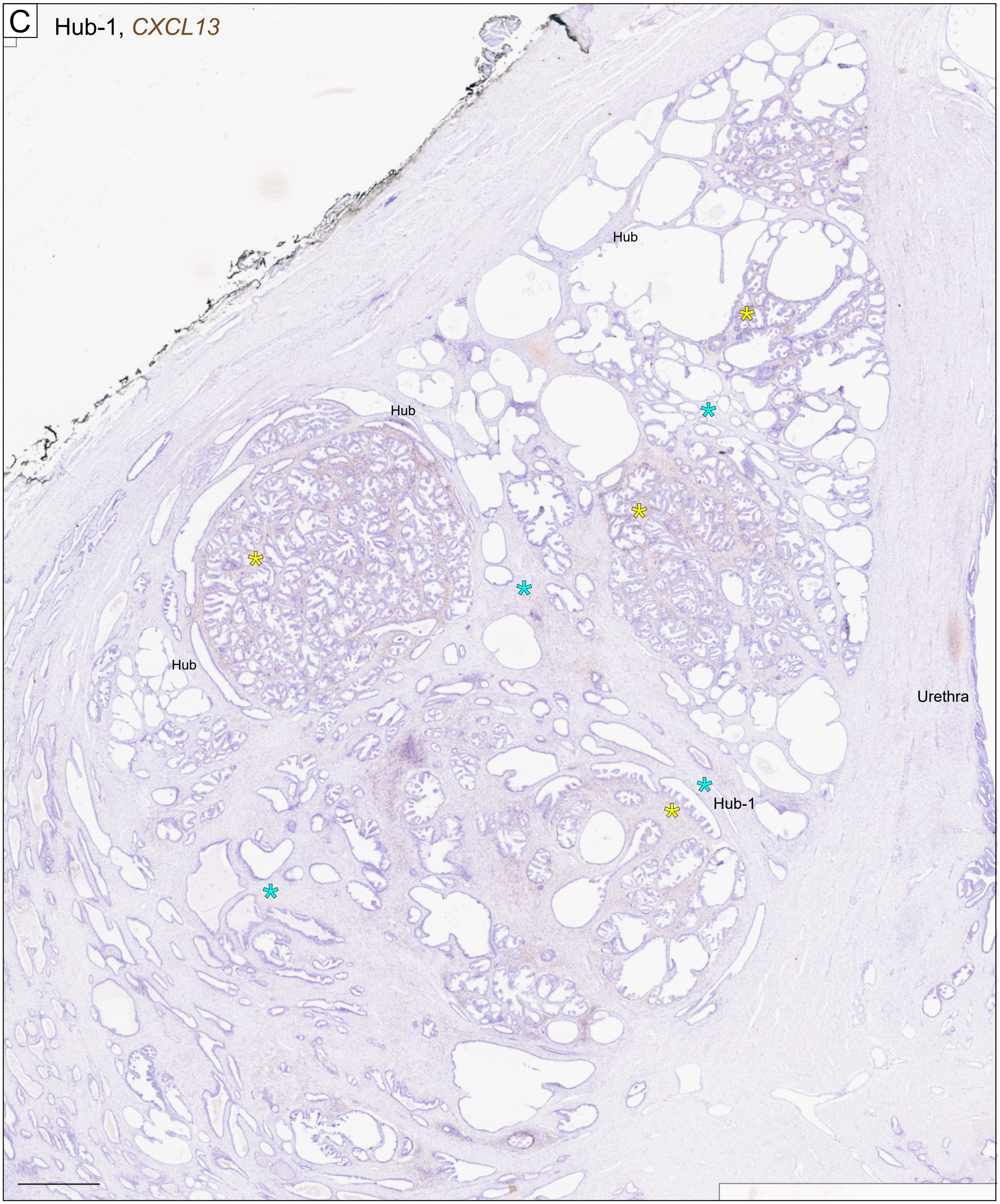

Supplemental Figure 2

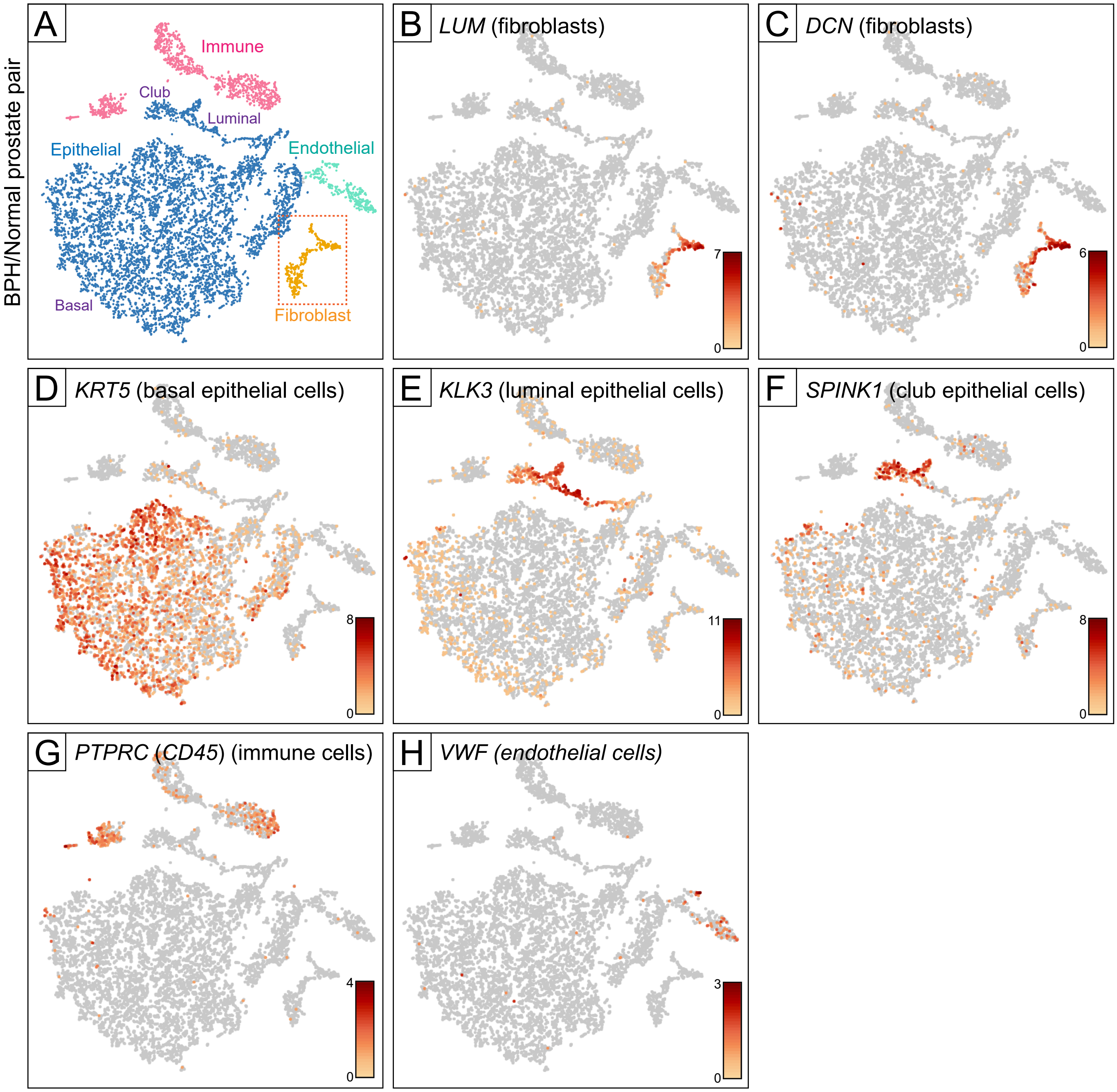

S**upplemental Figure 2. Single-cell RNAseq of prostate identifies BPH fibroblast cluster. (A)** t-SNE plot of combined BPH/normal prostate cells. Each dot represents an individual cell. Cell clusters (colored) are annotated by the expression of known cell-type markers. Note, this is the same panel as presented in Figure 4A.
(B-H) Expression of select known cell-type markers (color bar depicts log2 transcript counts per cell), including **(B)** *LUM* (fibroblasts), **(C)** *DCN* (fibroblasts), **(D)** *KRT5* (basal epithelial cells), **(E)** *KLK3* (luminal epithelial cells), **(F)** *SPINK1* (club cells), **(G)** *PTPRC* (*CD45*) (immune cells), and **(H)** *VWF* (endothelial cells).

Supplemental Figure 3

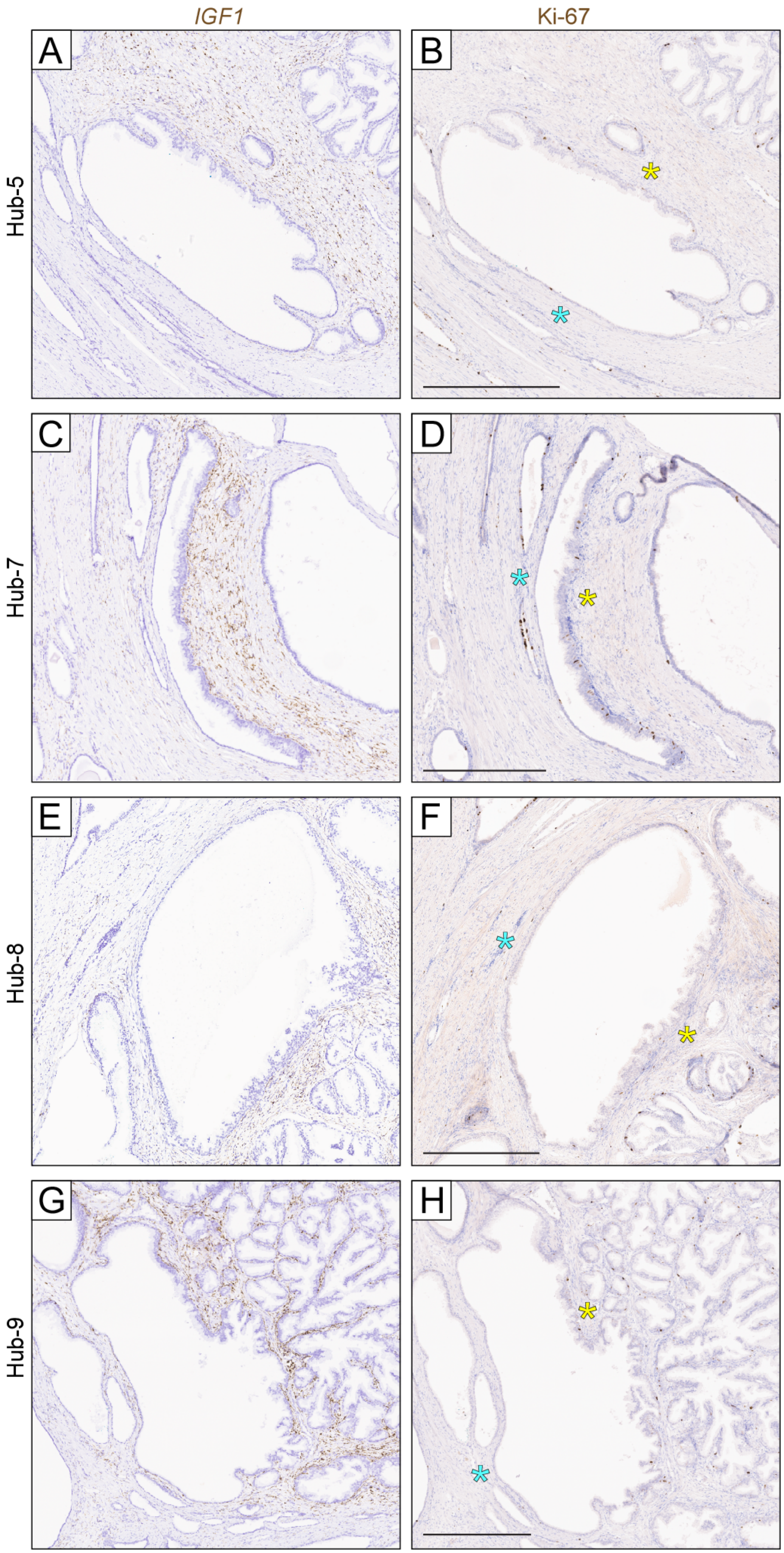

Supplemental Figure 3. Quantifying Ki-67+ ductal cells facing inner versus outer stroma. (A, B) Hub-5; **(C, D)** Hub-7; **(E, F)** Hub-8; and **(G, H)** Hub-9. **(A, C, E, G)** *IGF1* expression by RISH (brown staining). **(B, D, F, H)** Ki-67 staining (brown) by IHC. Ductal epithelial cells with Ki-67+ nuclear staining were separately counted on ductal wall facing the inner (*IGF1*+) versus outer (*IGF1*-) stroma. Scale bar is 500μM.

Supplemental Figure 4

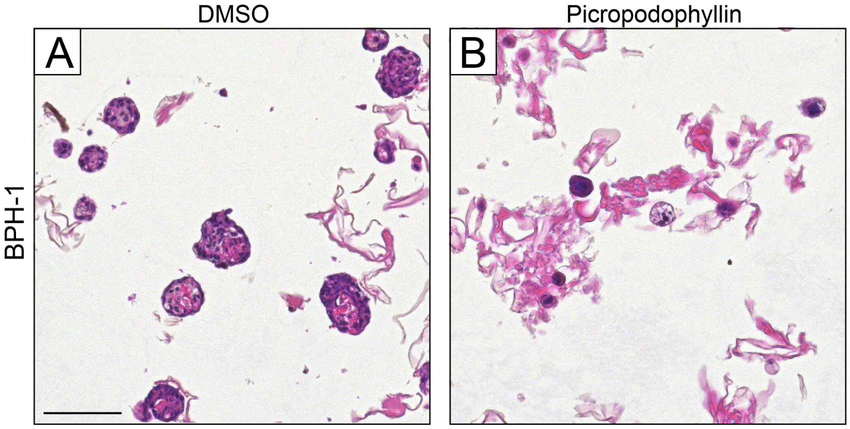

Supplemental Figure 4. Inhibition of IGF1 signaling thwarts BPH-1 spheroid formation. (A-B) H&E cross-sections of BPH-1 cell spheroids grown in Matrigel to 10 days, in media containing **(A)** vehicle (DMSO) or (**B**) IGF1R inhibitor Picropodophyllin (PPP) (1μM). Scale bar is 100 μM. Note that PPP treatment results in only single cells (but not spheroids) of varied size and nuclear morphology.

Supplemental Figure 5

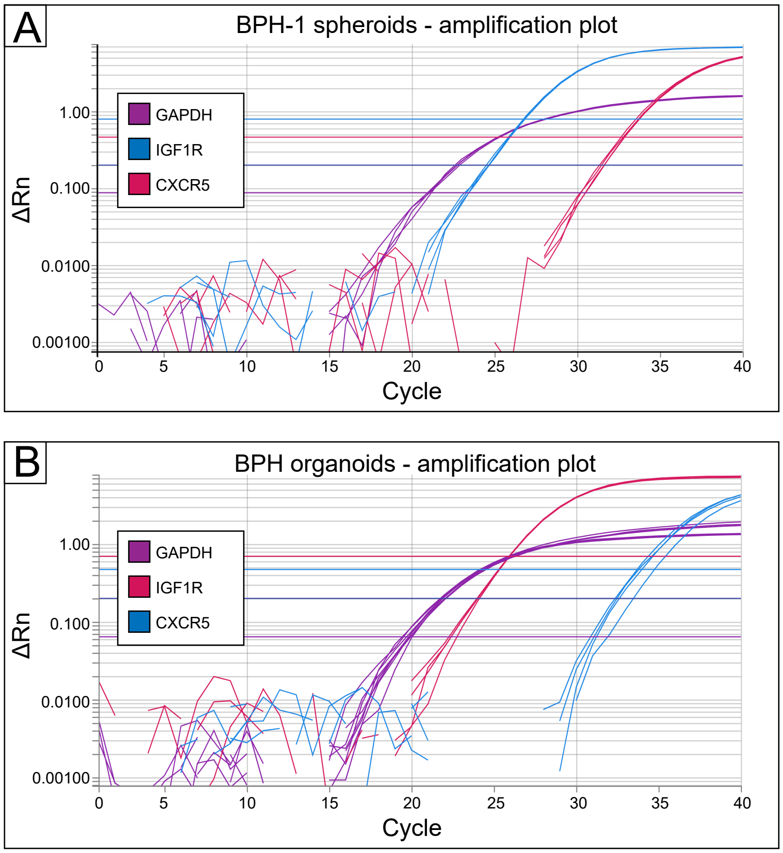

Supplemental Figure 5. *CXCR5* is expressed in BPH spheroids and organoids at substantially lower levels than *IGF1R*. (A-B) TaqMan q-RT-PCR amplification plots for **(A)** BPH-1 cell spheroids grown to 10 days, and **(B)** Patient-derived BPH organoids grown to 14 days. Note that the *CXCR5* amplification curve ascends 8-9 PCR cycles after the *IGF1R* curve, indicative of an approximate (since different gene probes are not quantitatively comparable) 2^8 (or 256) -fold lower expression level.
